# High-Throughput Proteomic Profiling to Evaluate Differentiation Syndrome With Menin Inhibition

**DOI:** 10.1016/j.mcpro.2026.101522

**Published:** 2026-01-30

**Authors:** Miriam B. Garcia, Bofei Wang, Irtiza Sheikh, Georgina El Hajjar, David McCall, Cesar Nunez, Amber Gibson, Philip L. Lorenzi, Ghayas C. Issa, Branko Cuglievan, Hussein A. Abbas

**Affiliations:** 1 Department of Pediatrics, The University of Texas MD Anderson Cancer Center, Houston, Texas, USA; 2 Department of Leukemia, The University of Texas MD Anderson Cancer Center, Houston, Texas, USA; 3 Department of Bioinformatics and Computational Biology, The University of Texas MD Anderson Cancer Center, Houston, Texas, USA; 4 Department of Genomic Medicine, The University of Texas MD Anderson Cancer Center, Houston, Texas, USA

**Keywords:** proteomics, pediatric, differentiation syndrome, menin inhibitors, relapsed refractory AML, acute myeloid leukemia

## Abstract

High-throughput proteomic profiling provides a comprehensive analysis of systemic cancer effects and tumor microenvironment interactions. Characterizing soluble proteins driving inflammation in acute myeloid leukemia (AML) offers insight into inflammatory diseases like differentiation syndrome related to AML therapies like menin inhibitors. We present our application of nucleic acid-linked immuno-sandwich assay, a novel technology leveraging next-generation sequencing for high-throughput, ultrasensitive characterization of secreted inflammatory proteins in plasma or serum. Here, we report its use to identify dynamic soluble protein changes during treatment and at the time of suspected differentiation syndrome in pediatric AML patients treated with the menin inhibitor revumenib (NCT04065399 and NCT05360160).

## Highlights

• Menin inhibitors are novel targeted therapies for rare-mutated acute myeloid leukemia.
• Differentiation syndrome and cytokine imbalance related to menin inhibition may be lethal.
• NULISAseq analysis of protein markers of differentiation syndrome may improve outcomes.
• NULISAseq may distinguish differentiation biomarkers from leukemia to guide treatment response.

## Graphical Abstract

**Figure:**
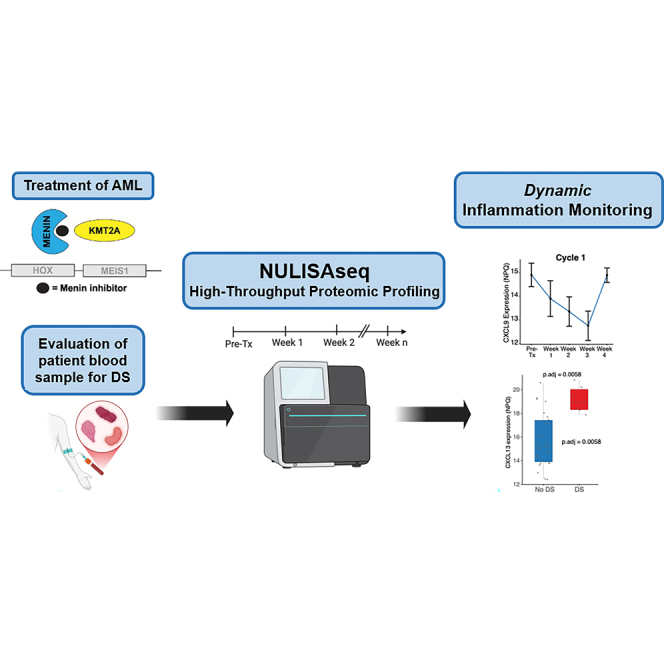

## In Brief

High-throughput proteomics using NULISAseq enables ultrasensitive detection of inflammatory proteins in acute myeloid leukemia. This NGS-based assay was utilized to identify dynamic protein changes during treatment and suspected differentiation syndrome in pediatric patients with acute myeloid leukemia receiving the menin inhibitor revumenib.

Menin inhibitors are novel targeted therapies for acute leukemia, particularly *KMT2A*- (*KMT2Ar*) or *NUP98*-rearranged (*NUP98r*) or *NPM1*-mutated subtypes. Menin inhibition leads to downregulation of the *HOX/MEIS1* transcriptional program necessary for leukemogenesis, therefore leading to hematopoietic differentiation and an antileukemic effect (1). The menin inhibitor revumenib was recently approved for relapsed or refractory acute leukemia with *KMT2Ar* in adult and pediatric patients (2). This was based on the results of AUGMENT-101, an open-label, multicenter phase 1/2 trial where revumenib monotherapy led to complete remission or complete remission with partial hematologic recovery of 21.2% and a median duration of response of 6.4 months (2, 3, 4). Menin inhibitors have been associated with clinically apparent differentiation syndrome (DS), which has resulted in fatal outcomes in some rare cases. DS is likely caused by a cytokine imbalance associated with differentiation and maturation of leukemia cells, supporting the use of steroids, and/or holding menin inhibitor for DS control. However, uncontrolled inflammation could also be associated with leukemia progression (5, 6). Understanding its evolution during a patient’s treatment course may allow for distinguishing between treatment-versus leukemia-related inflammation and may provide insights into treatment-related morbidity and treatment response. Further, identifying mediators of menin inhibitor-related DS, through novel technologies such as nucleic acid-linked immuno-sandwich assay (NULISA) and next-generation sequencing (7, 8), may uncover therapeutic targets to mitigate its complications.

## Experimental Procedures and Results

We applied NULISA to measure 249 inflammatory soluble proteins in 52 samples (Supplemental Table S1) of either serum or plasma from 15 pediatric patients (Table 1) with acute myeloid leukemia (AML) treated with revumenib. Samples were serum or plasma based on the biospecimen bank from which they were retrieved due to the retrospective nature of the analysis. Seven samples were collected when DS was clinically suspected in five patients. The median enrollment age for revumenib treatment was 11 years (range, 1–19) and 53.3% were male. Genetic alterations included *KMT2Ar* (*n* = 11, 73.3%), *NUP98r* (n = 3, 20%), and *NPM1* mutation (n = 1, 6.7%). Five of the 15 patients (33%) had clinical suspicion of DS warranting treatment with steroids with a median suspected onset of 7 days (range, 3–11) after starting revumenib. When DS diagnostic criteria were applied based on Montesinos *et al*, (9) two of the five patients were classified as moderate and one as severe DS (Supplemental Table S2).

**Table 1:** Baseline patient characteristics

**Patient characteristics**	Suspected DS (n = 5)	No DS (n = 10)	Total patients (N = 15)
Age, years (range)	11 (4–16)	13.5 (4–17)	11 (1–19)
Sex			
Female, n (%)	2 (40%)	5 (50%)	7 (47%)
Male, n (%)	3 (60%)	5 (50%)	8 (53%)
Race/Ethnicity			
Black, n (%)	0 (0%)	1 (10%)	1 (7%)
Hispanic, n (%)	2 (40%)	4 (40%)	6 (40%)
White (non-Hispanic), n (%)	3 (60%)	5 (50%)	8 (53%)
Genetic alteration			
*KMT2A*r, n (%)	5 (100%)	6 (60%)	11 (73%)
*NUP98*r, n (%)	0 (0%)	3 (30%)	3 (20%)
*NPM1mt*, n (%)	0 (0%)	1 (10%)	1 (7%)
WBC, median (range), K/uL	1.1 (0.7–11.5)	2.45 (0–9.7)	2.1 (0–11.5)
Hemoglobin, median (range), g/dl	8.4 (6.6–10.5)	8.9 (0–12)	8.5 (0–12)
Platelets, median (range), K/uL	16 (14–29)	27.5 (10.6–313)	19 (10.6–313)
Bone marrow blast %, median (range)	86 (77–97)	43 (4–88)	66% (4–97)
Peripheral blasts %, median (range)	7 (1–96)	6% (0–57)	6% (0–96)

DS, differentiation syndrome; WBC, white blood cells.

**Fig. 1:**
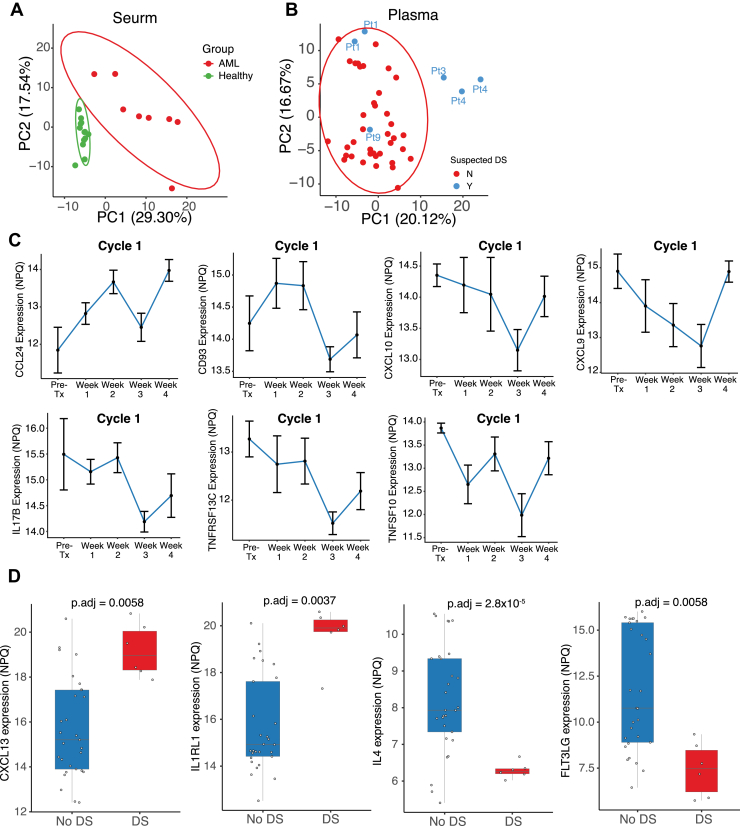
**Analysis of protein and cytokine expression of samples.***A*, principal component analysis (PCA) of protein expression for serum samples. Healthy samples and AML samples are clearly separated. *B*, principal component analysis (PCA) of protein expression for plasma samples. Three separated samples were drawn during a period of suspected DS. *C*, line graph demonstrating significant changes in the expression of cytokines. Samples were collected at pretreatment, week 1 (day 1–8; ± 2 days), week 2 (day 15; ± 2 days), week 3 (day 22; ± 2 days), and week 4 (day 29; ± 2 days) of cycle 1. Protein expressions demonstrate a significant pattern of change during menin inhibition across five groups based on treatment phase, (Kruskal test, *p* < 0.05). *D*, differential cytokine expression between patients who experienced DS compared to those without DS in plasma samples. Boxplot demonstrates significant cytokine expression in patients who did not experience DS (*blue*) compared to patients who did experience DS (*red*) during treatment with menin inhibition. Four cytokines were significantly altered in expression during DS including CXCL13, IL1RL1, IL4, and FLT3LG. The adjusted *p* values (p-adj) displayed above each plot denote the significance of the differences in expression between the two groups (p-adj < 0.01). AML, acute myeloid leukemia, DS, differentiation syndrome.

We found high detectability of the inflammatory proteins analyzed with only 2 of the 249 cytokines (IL36G and IL32) detected in less than 50% of samples (Supplemental Fig. S1, *A* and *B*). Due to the biological differences in plasma and serum, we analyzed samples from these sources separately. Principle component analysis of healthy (n = 13) and AML samples (n = 8) from serum clustered separately, with AML samples displaying more variability, suggesting the differences in proteomic profiling and intratumor heterogeneity in AML (Fig. 1*A*). AML plasma samples (n = 44) clustered together and contained six samples from four patients with suspected DS. Three samples which were drawn during DS notably separated from the cluster (Fig. 1*B*). Their separation indicated a distinct inflammatory pattern supporting our subsequent longitudinal analysis of samples collected at different treatment time points.

Among the 44 plasma samples, 35 were collected during cycle 1. We focused our analysis on the first cycle of revumenib, given that most cases of DS occur during this period (5). Longitudinal samples were categorized based on their weekly collection times, defined as: pretreatment (n = 4), week 1 (day 1–8; ± 2 days; n = 9), week 2 (day 15; ± 2 days; n = 7), week 3 (day 22; ± 2 days; n = 9) and week 4 (day 29; ± 2 days; n = 6). Expression of seven proteins (CCL24, CD93, CXCL10, CXCL9, IL17B, TNFRSF13C, and TNFSF10) significantly changed along the treatment course (Kruskal test, *p* < 0.05) (Fig. 1*C*; Supplemental Table S3). We found a decrease in levels of CXCL10 and CXCL9 during the first 3 weeks of cycle 1 followed by an increase during week 4 (Fig. 1*C*). The reduction of these two markers over the course of cycle 1 may explain a potential demarginalization or redistribution of leukemia cells from their environment following menin inhibition. Although other proteins showed distinct patterns, they all reached almost the lowest level at day 22 of cycle 1 (Fig. 1*C*), indicating the maximum drug effect at this point.

We next aimed to delineate the soluble proteins with levels significantly changed in patients who had clinical suspicion of DS requiring intervention with steroid treatment (n = 4 patients for six plasma samples) versus patients without clinical evidence of DS across all time points. Among the 249 soluble proteins evaluated, the expression levels of four cytokines (CXCL13, IL1RL1, IL4, and FLT3LG) were significantly different (p-adj < 0.01) between these two groups (Fig. 1*D*; Supplemental Table S4). CXCL13 and IL1RL1 were highly expressed in patients who had suspected DS, while IL4 and FLT3LG expression was significantly lower during that same period (Fig. 1*D*).

## Experimental Design and Statistical Rationale

The study was conducted in accordance with the Declaration of Helsinki and the use of human material was approved by MD Anderson’s Institutional Review Board. All data and records were kept confidential and deidentified, and biospecimens were received deidentified, and coded in accordance with institutional policies and the Health Insurance Portability and Accountability Act (HIPAA) on subject privacy.

We retrospectively analyzed 52 serum or plasma samples from 15 pediatric AML patients treated with revumenib using NULISAseq Inflammation Panel 250 (Alamar Biosciences) to quantify 249 inflammatory proteins. Samples were grouped by treatment week during cycle 1. Due to the biological differences between two sample sources and batch effects in different runs, we separated analysis for serum and plasma samples. Principal component analysis revealed distinct clustering of DS-associated samples. Temporal protein changes were assessed using the Kruskal–Wallis test, identifying seven proteins with significant variation (*p* < 0.05). Differential expression analysis comparing DS-suspected versus non-DS patients identified four significantly altered cytokines (adjusted *p* < 0.01). All statistical analyses were conducted using nonparametric methods due to the small sample size and expected non-normal distribution of protein expression levels. This design allowed us to explore both temporal and clinical phenotype-associated changes in the inflammatory proteome during revumenib treatment.

## Discussion

Our work supports the use of next-generation sequencing-based measures to detect low-expressing soluble proteins that can be overlooked with conventional protein assays. A unique inflammatory pattern was discovered in patient samples drawn during DS. Longitudinal sampling during the first cycle of therapy with menin inhibition showed a decrease in levels of CXCL10 and CXCL9. Interestingly, CXCL10 and CXCL9 can induce leukemic cell chemotaxis, enhance leukemic cell viability, and drug resistance (10, 11).

The changes in soluble proteins indicate distinct pathways in cytokine secretion in patients with AML who experienced DS due to menin inhibition. Though our limited dataset and analysis do not confirm the effect of the cytokine activity on leukemic cell maintenance and propagation in patients with AML and DS, some of the overexpressed cytokines have been described as playing a role in AML cell invasion and maintenance and the immune environment. In addition, our study provides feasibility to incorporate longitudinal soluble proteomic profiling in patients while on clinical trials. CXCL13 has also been described in signal modulation within the tumor microenvironment, promoting cancer cell invasion (12). Overexpression of IL1RL1 is a plausible mechanism for cytokine production and leukemic cell maintenance in AML (13). Thus, downregulation of IL1RL1 may be indicative of suppressed AML cell maintenance activity, a function of menin inhibition. Conversely, IL4 has been described to promote apoptosis and phagocytosis (14, 15), and its lower expression supports the rise in cell counts that occur during DS as blasts differentiate into mature cells. FLT3LG is associated with significant impairment of the immune system, and decreased levels of FLT3LG during DS could indicate consumption by activation of FLT3 during this process (16). The dosing of dexamethasone in patients with DS should also be taken into consideration with future analysis of cytokines as dexamethasone could drive their reduction, which may be seen with clinical correlation of therapeutic effect.

Although our study is limited by sample size due to the rarity of this disease and the applicability of revumenib in a distinct pediatric AML population, we provide proof-of-concept to detect soluble proteins associated with the phenomenon known as DS, which is a complication in this therapy. Our study is also powered by the longitudinal profiling of 52 samples from this patient cohort (n = 15) during treatment on cycle 1 with revumenib. Future prospective studies will allow for baseline sample collection prior to initiation of treatment for all patients analyzed to further characterize cytokine expression in DS and whether findings reflect an intrinsically different leukemia.

Through comprehensive proteomic profiling of inflammatory cytokines in pediatric patients receiving menin inhibition, we identified soluble proteins that correlate with DS. Our sample size necessitates further studies to validate the therapeutic significance of these findings. However, we demonstrated the feasibility of applying high-throughput soluble proteomics in AML therapy to identify biomarkers for preemptive management of DS, thus setting the stage for implementing protein analysis in longitudinal studies to assess inflammatory states objectively. Equally important, accurately identifying differentiation as the cause for signs and symptoms of a cytokine storm, as opposed to disease progression, may prevent early discontinuation of menin inhibition. Further identification of patterns associated with cytokines in patients with DS not found in patients who do not develop DS may determine protein mediators that may be targeted to mitigate its complications without necessitating medication holds that could delay time to remission or even effect outcomes negatively. On the contrary, patients with progressive disease have similar symptoms as DS and remaining on a menin inhibitor that is not adequately remitting disease could delay time to a more suitable agent, increasing risk of rapid progression and decreasing probability of survival.

Assessing the therapeutic potential of selected proteins will also be crucial and will require validation in preclinical models or organoid systems. Despite these challenges, our study demonstrates that high-throughput proteomics offers a robust and efficient approach to identifying novel and potent biomarkers for treatment with menin inhibitors and DS. Future work will expand proteomic profiling beyond 249 soluble proteins, applying it prospectively in larger AML and hematologic disease cohorts. Integration into clinical trials will evaluate its potential to guide therapy and improve outcomes.

## Data Availability

Patient level data can be available upon reasonable request from the corresponding authors (M. B. G or H. A. A.).

### Supplemental Data

This article contains supplemental data (9).

#### Ethics Approval

The study was conducted in accordance with the Declaration of Helsinki and approved by the Institutional Review Board MD Anderson Cancer Center.

## Conflict of Interest

G. C. I. received consulting or advisory role fees from Novartis, Kura Oncology, Syndax, NuProbe, AbbVie, Sanofi, AstraZeneca; received research funding from Novartis, Syndax, Kura Oncology, Merck, Cullinan Oncology, Astex Pharmaceuticals, NuProbe, Cullinan Oncology; received stock or other ownership from Pupil Bio. H. A. A. received Honoraria from Illumina and Alamar Biotechnology, in-kind support from Illumina, research support from Genentech, Enzyme-By-Design, GlaxoSmithKline, Blueprint Medicines, Ascentage and Illumina; served on advisory board for Cogent Biosciences and Consultant to Molecular Partners. M. B. G. has received honoraria for editorial writing for CMedEd, Pittman Communications. B. C. has received travel support from Octapharma and Syndax Pharmaceuticals. The other authors declare no competing interests.
