# Supplementary material for: High-Throughput Proteomic Profiling to Evaluate Differentiation Syndrome With Menin Inhibition
Source: Mol Cell Proteomics. 2026 Jan 30;25(3):101522. doi: 10.1016/j.mcpro.2026.101522 (PMC12955094; doi:10.1016/j.mcpro.2026.101522)

**Supplemental Tables**

**Table S1: Sample Characteristics**

| **Patient** | **Component (plasma/serum)** | **Collection Timepoint^** |
| --- | --- | --- |
| 1 | plasma | 2 |
|  | plasma | 3 |
|  | plasma | 4 |
| **2** | plasma | 1 |
|  | plasma | 2 |
|  | plasma | 4 |
|  | plasma | 6 |
|  | serum | 6 |
|  | serum | 8 |
|  | plasma | 7 |
| **3** | plasma | 2 |
|  | plasma | 4 |
| **4** | plasma | 2 |
|  | plasma | 3 |
| **5** | plasma | 4 |
|  | plasma | 5 |
|  | plasma | 6 |
| **6** | plasma | 3 |
|  | plasma | 5 |
| **7** | plasma | 2 |
|  | plasma | 3 |
|  | plasma | 4 |
|  | plasma | 5 |
|  | plasma | 6 |
|  | plasma | 6 |
| **8** | plasma | 2 |
|  | plasma | 3 |
|  | plasma | 4 |
|  | plasma | 5 |
| **9** | plasma | 2 |
|  | plasma | 5 |
|  | plasma | 8 |
|  | plasma | 8 |
| **10** | plasma | 1 |
|  | serum | 2 |
|  | plasma | 2 |
|  | plasma | 3 |
|  | plasma | 4 |
|  | plasma | 7 |
| **11** | plasma | 1 |
|  | plasma | 2 |
|  | plasma | 4 |
|  | plasma | 6 |
| **12** | plasma | 1 |
|  | plasma | 3 |
|  | plasma | 4 |
|  | plasma | 5 |
| **13** | serum | 1 |
|  | serum | 6 |
| **14** | serum | 7 |
| **15** | serum | 1 |
|  | serum | 5 |

^Collection Timepoint: 1 - Pretreatment, 2 – cycle 1, week 1 (day 1-8; +/-2 days), 3 - cycle 1, week 2 (day 15; +/-2 days), 4 - cycle 1, week 3 (day 22; +/-2 days), 5 - cycle 1, week 4 (day 29; +/-2 days), 6 – cycles prior to transplant excluding cycle 1, 7 – post transplant on menin inhibitor, 8 – post transplant off menin inhibitor

**Table S2: Characterization of suspected differentiation syndrome**

| **Patient** | **Onset^** | **Signs/Symptoms^** | **Grading* (moderate/severe)** | **Duration of treatment with dexamethasone** |
| --- | --- | --- | --- | --- |
| 1 | C1 D10 | edema, rash | - | 14 days |
| 3 | C1 D11 | edema, rash, lung infiltrate | moderate | 6 days |
| 4 | C1 D4 | dyspnea, weight gain, AKI, pleural and pericardial effusion, edema, lung infiltrates | severe | 8 days |
| 9 | C1 D7 | edema, rash | - | 10 days |
| 15 | C1 D3 | fever, pericardial effusion, lung infiltrates, edema | moderate | 6 days |

^Clinically suspected differentiation syndrome including any sign or symptom beyond those listed in the Montesinos criteria, with day of onset during cycle 1 denoted.

*As defined by the Montesinos criteria, where 2 or more signs or symptoms among the following are needed for a differentiation syndrome diagnosis: fever, weight gain of ≥5kg, hypotension, lung infiltrates, pleural effusion, pericardial effusion, edema. Grading is defined as ‘moderate’ if 2-3 signs/symptoms and ‘severe’ if 4 or more signs/symptoms. AKI: acute kidney injury; C: cycle; D: day.

**Table S3: P-value for longitudinal change of proteins**

| **cytokine** | **pval** | **padjust** |
| --- | --- | --- |
| AGER | 0.108802 | 0.83188801 |
| AGRP | 0.60698118 | 0.84676731 |
| ANGPT1 | 0.64432452 | 0.84825579 |
| ANGPT2 | 0.59929896 | 0.84676731 |
| ANXA1 | 0.4543788 | 0.83188801 |
| AREG | 0.506672 | 0.83188801 |
| BDNF | 0.43708223 | 0.83188801 |
| BMP7 | 0.476512 | 0.83188801 |
| BST2 | 0.36639064 | 0.83188801 |
| C1QA | 0.6462878 | 0.84825579 |
| CALCA | 0.55793292 | 0.83188801 |
| CCL1 | 0.57973472 | 0.84282876 |
| CCL11 | 0.3333148 | 0.83188801 |
| CCL13 | 0.42104692 | 0.83188801 |
| CCL14 | 0.20527016 | 0.83188801 |
| CCL15 | 0.8271769 | 0.9154091 |
| CCL16 | 0.42887688 | 0.83188801 |
| CCL17 | 0.24980103 | 0.83188801 |
| CCL19 | 0.26957465 | 0.83188801 |
| CCL2 | 0.70772835 | 0.85963102 |
| CCL20 | 0.32162273 | 0.83188801 |
| CCL21 | 0.09470608 | 0.83188801 |
| CCL22 | 0.17564727 | 0.83188801 |
| CCL23 | 0.97957547 | 0.98352537 |
| CCL24 | 0.01149859 | 0.83188801 |
| CCL25 | 0.93381 | 0.95686704 |
| CCL26 | 0.25697964 | 0.83188801 |
| CCL27 | 0.3021305 | 0.83188801 |
| CCL28 | 0.24124514 | 0.83188801 |
| CCL3 | 0.47099338 | 0.83188801 |
| CCL4 | 0.31196697 | 0.83188801 |
| CCL5 | 0.47064869 | 0.83188801 |
| CCL7 | 0.73225532 | 0.87127058 |
| CCL8 | 0.29168004 | 0.83188801 |
| CD200 | 0.53030705 | 0.83188801 |
| CD200R1 | 0.08859968 | 0.83188801 |
| CD27 | 0.11644108 | 0.83188801 |
| CD274 | 0.7030235 | 0.858415 |
| CD276 | 0.67048164 | 0.85178534 |
| CD3E | 0.30928953 | 0.83188801 |
| CD4 | 0.26455869 | 0.83188801 |
| CD40 | 0.51224438 | 0.83188801 |
| CD40LG | 0.46480198 | 0.83188801 |
| CD46 | 0.06514558 | 0.83188801 |
| CD70 | 0.07281947 | 0.83188801 |
| CD80 | 0.63942584 | 0.84825579 |
| CD83 | 0.09168347 | 0.83188801 |
| CD93 | 0.04578453 | 0.83188801 |
| CEACAM5 | 0.94910628 | 0.96855518 |
| CHI3L1 | 0.79666072 | 0.91070953 |
| CLEC4A | 0.17308131 | 0.83188801 |
| CNTF | 0.62946985 | 0.84825579 |
| CRP | 0.47472482 | 0.83188801 |
| CSF1 | 0.59777065 | 0.84676731 |
| CSF1R | 0.42123087 | 0.83188801 |
| CSF2 | 0.68729942 | 0.85710534 |
| CSF2RB | 0.44831834 | 0.83188801 |
| CSF3 | 0.53657544 | 0.83188801 |
| CSF3R | 0.39491048 | 0.83188801 |
| CST7 | 0.61037243 | 0.84676731 |
| CTF1 | 0.61212094 | 0.84676731 |
| CTLA4 | 0.17842386 | 0.83188801 |
| CTSS | 0.86830809 | 0.93596846 |
| CX3CL1 | 0.93322746 | 0.95686704 |
| CXADR | 0.42176203 | 0.83188801 |
| CXCL1 | 0.40489709 | 0.83188801 |
| CXCL10 | 0.0418077 | 0.83188801 |
| CXCL11 | 0.66864563 | 0.85178534 |
| CXCL12 | 0.43527063 | 0.83188801 |
| CXCL13 | 0.46032279 | 0.83188801 |
| CXCL14 | 0.83809688 | 0.91695041 |
| CXCL16 | 0.92760407 | 0.95686704 |
| CXCL2 | 0.30714322 | 0.83188801 |
| CXCL3 | 0.65079775 | 0.84842219 |
| CXCL5 | 0.69346343 | 0.85710534 |
| CXCL6 | 0.52996316 | 0.83188801 |
| CXCL8 | 0.3283217 | 0.83188801 |
| CXCL9 | 0.0366951 | 0.83188801 |
| EGF | 0.39952387 | 0.83188801 |
| EPO | 0.87303954 | 0.93701226 |
| FASLG | 0.54135667 | 0.83188801 |
| FGF19 | 0.55501493 | 0.83188801 |
| FGF2 | 0.54142507 | 0.83188801 |
| FGF21 | 0.64726346 | 0.84825579 |
| FGF23 | 0.16468564 | 0.83188801 |
| FLT1 | 0.43141225 | 0.83188801 |
| FLT3LG | 0.36635282 | 0.83188801 |
| FLT4 | 0.05189076 | 0.83188801 |
| FTH1 | 0.72533979 | 0.87127058 |
| FURIN | 0.39342499 | 0.83188801 |
| GDF15 | 0.48718448 | 0.83188801 |
| GDF2 | 0.76316356 | 0.89214896 |
| GFAP | 0.5277312 | 0.83188801 |
| GRN | 0.05742644 | 0.83188801 |
| GZMA | 0.263292 | 0.83188801 |
| GZMB | 0.55340172 | 0.83188801 |
| HAVCR1 | 0.32920172 | 0.83188801 |
| HGF | 0.137426 | 0.83188801 |
| HLA_DRA | 0.65817888 | 0.84915307 |
| ICAM1 | 0.14319018 | 0.83188801 |
| ICOSLG | 0.35093677 | 0.83188801 |
| IFNA1_IFNA13 | 0.61906375 | 0.84825579 |
| IFNA2 | 0.39640525 | 0.83188801 |
| IFNB1 | 0.33479035 | 0.83188801 |
| IFNG | 0.21111195 | 0.83188801 |
| IFNL1 | 0.45847696 | 0.83188801 |
| IFNL2_IFNL3 | 0.51454438 | 0.83188801 |
| IFNW1 | 0.05282276 | 0.83188801 |
| IKBKG | 0.3636382 | 0.83188801 |
| IL10 | 0.80225701 | 0.91070953 |
| IL10RB | 0.4015997 | 0.83188801 |
| IL11 | 0.63724787 | 0.84825579 |
| IL12B | 0.38825684 | 0.83188801 |
| IL12p70 | 0.77924417 | 0.90247348 |
| IL12RB1 | 0.26653943 | 0.83188801 |
| IL13 | 0.42567396 | 0.83188801 |
| IL13RA2 | 0.81556806 | 0.91070953 |
| IL15 | 0.86094577 | 0.93206738 |
| IL15RA | 0.34031718 | 0.83188801 |
| IL16 | 0.83961724 | 0.91695041 |
| IL17A | 0.38580056 | 0.83188801 |
| IL17A_IL17F | 0.17035525 | 0.83188801 |
| IL17B | 0.04366741 | 0.83188801 |
| IL17C | 0.96509501 | 0.97626834 |
| IL17F | 0.91211363 | 0.95686704 |
| IL17RA | 0.20555737 | 0.83188801 |
| IL17RB | 0.5841595 | 0.84282876 |
| IL18 | 0.73480651 | 0.87127058 |
| IL18BP | 0.30064523 | 0.83188801 |
| IL18R1 | 0.56776523 | 0.84150918 |
| IL19 | 0.81561537 | 0.91070953 |
| IL1B | 0.23656198 | 0.83188801 |
| IL1R1 | 0.88638854 | 0.94021034 |
| IL1R2 | 0.88734711 | 0.94021034 |
| IL1RL1 | 0.32122267 | 0.83188801 |
| IL1RN | 0.4939207 | 0.83188801 |
| IL2 | 0.14120808 | 0.83188801 |
| IL20 | 0.41676984 | 0.83188801 |
| IL22 | 0.75398531 | 0.88575649 |
| IL23 | 0.66481389 | 0.85178534 |
| IL24 | 0.07826074 | 0.83188801 |
| IL27 | 0.83544661 | 0.91695041 |
| IL2RA | 0.52462579 | 0.83188801 |
| IL2RB | 0.10619869 | 0.83188801 |
| IL32 | 0.2201374 | 0.83188801 |
| IL33 | 0.2012466 | 0.83188801 |
| IL34 | 0.6953224 | 0.85710534 |
| IL36A | 0.32810092 | 0.83188801 |
| IL36B | 0.72056689 | 0.87097648 |
| IL36G | 0.21481077 | 0.83188801 |
| IL3RA | 0.15065411 | 0.83188801 |
| IL4 | 0.47525706 | 0.83188801 |
| IL4R | 0.0992361 | 0.83188801 |
| IL5 | 0.29151513 | 0.83188801 |
| IL5RA | 0.35426159 | 0.83188801 |
| IL6 | 0.72820995 | 0.87127058 |
| IL6R | 0.67467952 | 0.85276752 |
| IL6ST | 0.23436415 | 0.83188801 |
| IL7 | 0.63236291 | 0.84825579 |
| IL7R | 0.42032747 | 0.83188801 |
| IL9 | 0.55157745 | 0.83188801 |
| IRAK4 | 0.371801 | 0.83188801 |
| KDR | 0.23629612 | 0.83188801 |
| KITLG | 0.93191576 | 0.95686704 |
| KLRK1 | 0.25303064 | 0.83188801 |
| KNG1 | 0.06242892 | 0.83188801 |
| LAG3 | 0.10093148 | 0.83188801 |
| LAMP3 | 0.18243687 | 0.83188801 |
| LCN2 | 0.80535787 | 0.91070953 |
| LGALS9 | 0.122862 | 0.83188801 |
| LIF | 0.79984718 | 0.91070953 |
| LILRB2 | 0.7685527 | 0.89425057 |
| LTA | 0.44512087 | 0.83188801 |
| LTA_LTB | 0.98623404 | 0.98623404 |
| MERTK | 0.25048688 | 0.83188801 |
| MICA | 0.43836873 | 0.83188801 |
| MICB | 0.36296202 | 0.83188801 |
| MIF | 0.69295222 | 0.85710534 |
| MMP1 | 0.84900174 | 0.92315037 |
| MMP12 | 0.81158791 | 0.91070953 |
| MMP3 | 0.4002166 | 0.83188801 |
| MMP8 | 0.95323513 | 0.96879815 |
| MMP9 | 0.80877855 | 0.91070953 |
| MPO | 0.9011699 | 0.95081062 |
| MUC16 | 0.49245954 | 0.83188801 |
| NAMPT | 0.43464108 | 0.83188801 |
| NCR1 | 0.4432909 | 0.83188801 |
| NGF | 0.75413805 | 0.88575649 |
| NTF3 | 0.41651955 | 0.83188801 |
| OSM | 0.96842682 | 0.97626834 |
| OSMR | 0.54865946 | 0.83188801 |
| PDCD1 | 0.17777767 | 0.83188801 |
| PDCD1LG2 | 0.14687026 | 0.83188801 |
| PDGFA | 0.54148103 | 0.83188801 |
| PDGFB | 0.51043664 | 0.83188801 |
| PGF | 0.2396232 | 0.83188801 |
| PTX3 | 0.57426667 | 0.84282876 |
| S100A12 | 0.82493971 | 0.9154091 |
| S100A9 | 0.46701604 | 0.83188801 |
| SCG2 | 0.64271489 | 0.84825579 |
| SDC1 | 0.44950711 | 0.83188801 |
| SELE | 0.54694418 | 0.83188801 |
| SELP | 0.45827591 | 0.83188801 |
| SIRPA | 0.14270481 | 0.83188801 |
| SLAMF1 | 0.12868441 | 0.83188801 |
| SPP1 | 0.14304357 | 0.83188801 |
| TAFA5 | 0.25950166 | 0.83188801 |
| TEK | 0.08958318 | 0.83188801 |
| TGFB1 | 0.33327097 | 0.83188801 |
| TGFB3 | 0.59511858 | 0.84676731 |
| THBS2 | 0.33743395 | 0.83188801 |
| THPO | 0.58557982 | 0.84282876 |
| TIMP1 | 0.53148094 | 0.83188801 |
| TIMP2 | 0.10391025 | 0.83188801 |
| TLR3 | 0.18331831 | 0.83188801 |
| TNF | 0.3491358 | 0.83188801 |
| TNFRSF11A | 0.30794356 | 0.83188801 |
| TNFRSF11B | 0.70327976 | 0.858415 |
| TNFRSF13B | 0.25496529 | 0.83188801 |
| TNFRSF13C | 0.04905608 | 0.83188801 |
| TNFRSF14 | 0.24443988 | 0.83188801 |
| TNFRSF17 | 0.24188721 | 0.83188801 |
| TNFRSF18 | 0.65558385 | 0.84915307 |
| TNFRSF1A | 0.6889894 | 0.85710534 |
| TNFRSF1B | 0.26493862 | 0.83188801 |
| TNFRSF21 | 0.49174467 | 0.83188801 |
| TNFRSF4 | 0.17768844 | 0.83188801 |
| TNFRSF8 | 0.20521219 | 0.83188801 |
| TNFRSF9 | 0.26933222 | 0.83188801 |
| TNFSF10 | 0.02730131 | 0.83188801 |
| TNFSF11 | 0.12070646 | 0.83188801 |
| TNFSF12 | 0.12760207 | 0.83188801 |
| TNFSF13 | 0.33118112 | 0.83188801 |
| TNFSF14 | 0.2617867 | 0.83188801 |
| TNFSF15 | 0.60477128 | 0.84676731 |
| TNFSF18 | 0.8828662 | 0.94021034 |
| TNFSF4 | 0.25767887 | 0.83188801 |
| TNFSF8 | 0.38472417 | 0.83188801 |
| TNFSF9 | 0.4503119 | 0.83188801 |
| TREM1 | 0.93253871 | 0.95686704 |
| TREM2 | 0.92882586 | 0.95686704 |
| VCAM1 | 0.50574702 | 0.83188801 |
| VEGFA | 0.5339444 | 0.83188801 |
| VEGFC | 0.15429544 | 0.83188801 |
| VEGFD | 0.4605917 | 0.83188801 |
| VSNL1 | 0.4165769 | 0.83188801 |
| VSTM1 | 0.63339117 | 0.84825579 |
| WNT16 | 0.11658144 | 0.83188801 |
| WNT7A | 0.58184794 | 0.84282876 |

**Table S4: P-value for DS vs Non-DS across all proteins**

| **cytokine** | **pval** | **padjust** |
| --- | --- | --- |
| AGER | 0.6232404 | 0.78775056 |
| AGRP | 0.47050208 | 0.68113382 |
| ANGPT1 | 0.98161899 | 0.98956732 |
| ANGPT2 | 0.06840413 | 0.32755057 |
| ANXA1 | 0.26404471 | 0.55025514 |
| AREG | 0.00152323 | 0.03816878 |
| BDNF | 0.40016086 | 0.65090796 |
| BMP7 | 0.18108395 | 0.43776606 |
| BST2 | 0.11981309 | 0.39569001 |
| C1QA | 0.00153288 | 0.03816878 |
| CALCA | 0.07566839 | 0.34188154 |
| CCL1 | 0.66490835 | 0.8041979 |
| CCL11 | 0.39989203 | 0.65090796 |
| CCL13 | 0.22124399 | 0.48130654 |
| CCL14 | 0.40685133 | 0.65090796 |
| CCL15 | 0.19610912 | 0.46398885 |
| CCL16 | 0.11839998 | 0.39569001 |
| CCL17 | 0.22229017 | 0.48130654 |
| CCL19 | 0.49488124 | 0.68864061 |
| CCL2 | 0.87903597 | 0.9323993 |
| CCL20 | 0.01472888 | 0.16665496 |
| CCL21 | 0.37353858 | 0.63272862 |
| CCL22 | 0.02398604 | 0.19418213 |
| CCL23 | 0.08475738 | 0.35435731 |
| CCL24 | 0.2651832 | 0.55025514 |
| CCL25 | 0.76438609 | 0.86514607 |
| CCL26 | 0.20603955 | 0.46398885 |
| CCL27 | 0.43050446 | 0.65090796 |
| CCL28 | 0.53008575 | 0.71346677 |
| CCL3 | 0.15341728 | 0.40875746 |
| CCL4 | 0.48479795 | 0.68864061 |
| CCL5 | 0.31052318 | 0.59023107 |
| CCL7 | 0.20816808 | 0.46398885 |
| CCL8 | 0.08425704 | 0.35435731 |
| CD200 | 0.12236197 | 0.39569001 |
| CD200R1 | 0.70440311 | 0.83126244 |
| CD27 | 0.35873056 | 0.63272862 |
| CD274 | 0.18314208 | 0.43848441 |
| CD276 | 0.43604006 | 0.65090796 |
| CD3E | 0.28728609 | 0.57003064 |
| CD4 | 0.14007869 | 0.40875746 |
| CD40 | 0.15431005 | 0.40875746 |
| CD40LG | 0.09051074 | 0.35773291 |
| CD46 | 0.00708099 | 0.11019798 |
| CD70 | 0.01568393 | 0.16665496 |
| CD80 | 0.86581585 | 0.9323993 |
| CD83 | 0.01673243 | 0.16665496 |
| CD93 | 0.05722003 | 0.31661752 |
| CEACAM5 | 0.51397687 | 0.69554479 |
| CHI3L1 | 0.54471351 | 0.71942858 |
| CLEC4A | 0.20870181 | 0.46398885 |
| CNTF | 0.63892039 | 0.79545589 |
| CRP | 0.61727472 | 0.78419084 |
| CSF1 | 0.26357562 | 0.55025514 |
| CSF1R | 0.508464 | 0.69461653 |
| CSF2 | 0.36430137 | 0.63272862 |
| CSF2RB | 0.92016415 | 0.95467519 |
| CSF3 | 0.87292028 | 0.9323993 |
| CSF3R | 0.11589866 | 0.39532558 |
| CST7 | 0.63285626 | 0.79545589 |
| CTF1 | 0.90524093 | 0.95467519 |
| CTLA4 | 0.20166328 | 0.46398885 |
| CTSS | 0.57446785 | 0.74115282 |
| CX3CL1 | 0.35514839 | 0.63165678 |
| CXADR | 0.13644973 | 0.40875746 |
| CXCL1 | 0.34450307 | 0.62614062 |
| CXCL10 | 0.28485842 | 0.57003064 |
| CXCL11 | 0.20055063 | 0.46398885 |
| CXCL12 | 0.10150293 | 0.3772273 |
| CXCL13 | 8.86E-05 | 0.00582104 |
| CXCL14 | 0.14819106 | 0.40875746 |
| CXCL16 | 0.43996177 | 0.65090796 |
| CXCL2 | 0.43541465 | 0.65090796 |
| CXCL3 | 0.15775931 | 0.41349546 |
| CXCL5 | 0.37242968 | 0.63272862 |
| CXCL6 | 0.10332471 | 0.37835079 |
| CXCL8 | 0.17416164 | 0.4293688 |
| CXCL9 | 0.37312044 | 0.63272862 |
| EGF | 0.3130763 | 0.59057574 |
| EPO | 0.11328335 | 0.39177158 |
| FASLG | 0.24353865 | 0.5183002 |
| FGF19 | 0.36459442 | 0.63272862 |
| FGF2 | 0.13187938 | 0.40875746 |
| FGF21 | 0.43527569 | 0.65090796 |
| FGF23 | 0.16761303 | 0.42157218 |
| FLT1 | 0.06161034 | 0.32213198 |
| FLT3LG | 9.35E-05 | 0.00582104 |
| FLT4 | 0.87162929 | 0.9323993 |
| FTH1 | 0.03363693 | 0.23642144 |
| FURIN | 0.74986334 | 0.85258435 |
| GDF15 | 0.01200021 | 0.16600289 |
| GDF2 | 0.15300893 | 0.40875746 |
| GFAP | 0.06525704 | 0.32213198 |
| GRN | 0.09959514 | 0.3772273 |
| GZMA | 0.87997525 | 0.9323993 |
| GZMB | 0.55334527 | 0.72517354 |
| HAVCR1 | 0.06212061 | 0.32213198 |
| HGF | 0.05311106 | 0.30224587 |
| HLA_DRA | 0.67628703 | 0.80959361 |
| ICAM1 | 0.20719301 | 0.46398885 |
| ICOSLG | 0.41800797 | 0.65090796 |
| IFNA1_IFNA13 | 0.27672245 | 0.56478598 |
| IFNA2 | 0.04561108 | 0.28392896 |
| IFNB1 | 0.5105013 | 0.69461653 |
| IFNG | 0.04168624 | 0.27315459 |
| IFNL1 | 0.37977695 | 0.63894905 |
| IFNL2_IFNL3 | 0.29718917 | 0.57364422 |
| IFNW1 | 0.40255466 | 0.65090796 |
| IKBKG | 0.84261715 | 0.92428048 |
| IL10 | 0.00104075 | 0.0323934 |
| IL10RB | 0.01277406 | 0.16665496 |
| IL11 | 0.29429971 | 0.57277515 |
| IL12B | 0.59782083 | 0.76730612 |
| IL12p70 | 0.3197468 | 0.59566344 |
| IL12RB1 | 0.15332124 | 0.40875746 |
| IL13 | 0.04936654 | 0.29981142 |
| IL13RA2 | 0.54075645 | 0.71942858 |
| IL15 | 0.0366083 | 0.24636395 |
| IL15RA | 0.11245269 | 0.39177158 |
| IL16 | 0.06430061 | 0.32213198 |
| IL17A | 0.04553366 | 0.28392896 |
| IL17A_IL17F | 0.49610562 | 0.68864061 |
| IL17B | 0.50057811 | 0.68864061 |
| IL17C | 0.15942727 | 0.41351448 |
| IL17F | 0.41708949 | 0.65090796 |
| IL17RA | 0.01986125 | 0.18316489 |
| IL17RB | 0.12986784 | 0.40875746 |
| IL18 | 0.63854067 | 0.79545589 |
| IL18BP | 0.45962557 | 0.66927934 |
| IL18R1 | 0.16420435 | 0.42151427 |
| IL19 | 0.30085652 | 0.57625594 |
| IL1B | 0.54607229 | 0.71942858 |
| IL1R1 | 0.79562376 | 0.88838707 |
| IL1R2 | 0.03283989 | 0.23642144 |
| IL1RL1 | 3.01E-05 | 0.00374437 |
| IL1RN | 0.11212346 | 0.39177158 |
| IL2 | 0.02417529 | 0.19418213 |
| IL20 | 0.78878985 | 0.88505276 |
| IL22 | 0.02389153 | 0.19418213 |
| IL23 | 0.49942831 | 0.68864061 |
| IL24 | 0.0853873 | 0.35435731 |
| IL27 | 0.85731877 | 0.9323993 |
| IL2RA | 0.17694401 | 0.43195155 |
| IL2RB | 0.9106426 | 0.95467519 |
| IL32 | 0.74427945 | 0.8518191 |
| IL33 | 0.28844924 | 0.57003064 |
| IL34 | 0.20868614 | 0.46398885 |
| IL36A | 0.27941347 | 0.5656419 |
| IL36B | 0.68208631 | 0.81100068 |
| IL36G | 0.26827046 | 0.5520607 |
| IL3RA | 0.43605251 | 0.65090796 |
| IL4 | 1.12E-07 | 2.78E-05 |
| IL4R | 0.65114754 | 0.79811935 |
| IL5 | 0.65388091 | 0.79811935 |
| IL5RA | 0.1306453 | 0.40875746 |
| IL6 | 0.64410336 | 0.79695411 |
| IL6R | 0.01963184 | 0.18316489 |
| IL6ST | 0.07858126 | 0.34327604 |
| IL7 | 0.42879321 | 0.65090796 |
| IL7R | 0.22535124 | 0.48372809 |
| IL9 | 0.14936295 | 0.40875746 |
| IRAK4 | 0.74314497 | 0.8518191 |
| KDR | 0.45762458 | 0.66927934 |
| KITLG | 0.03418141 | 0.23642144 |
| KLRK1 | 0.96504785 | 0.97683018 |
| KNG1 | 0.10031841 | 0.3772273 |
| LAG3 | 0.96506114 | 0.97683018 |
| LAMP3 | 0.82703601 | 0.91703601 |
| LCN2 | 0.95825649 | 0.97683018 |
| LGALS9 | 0.03076842 | 0.23642144 |
| LIF | 0.39738956 | 0.65090796 |
| LILRB2 | 0.07387645 | 0.34188154 |
| LTA | 0.66855006 | 0.8041979 |
| LTA_LTB | 0.57013892 | 0.73939891 |
| MERTK | 0.00509343 | 0.08852548 |
| MICA | 0.83232987 | 0.91703601 |
| MICB | 0.48036234 | 0.68741507 |
| MIF | 0.87827149 | 0.9323993 |
| MMP1 | 0.74387117 | 0.8518191 |
| MMP12 | 0.54473807 | 0.71942858 |
| MMP3 | 0.07688902 | 0.34188154 |
| MMP8 | 0.3677882 | 0.63272862 |
| MMP9 | 0.05340891 | 0.30224587 |
| MPO | 0.98639062 | 0.990368 |
| MUC16 | 0.55839169 | 0.72795566 |
| NAMPT | 0.78908318 | 0.88505276 |
| NCR1 | 0.95889106 | 0.97683018 |
| NGF | 0.05268871 | 0.30224587 |
| NTF3 | 0.15190408 | 0.40875746 |
| OSM | 0.95078538 | 0.97683018 |
| OSMR | 0.064758 | 0.32213198 |
| PDCD1 | 0.92335314 | 0.95467519 |
| PDCD1LG2 | 0.71663964 | 0.83776183 |
| PDGFA | 0.33544178 | 0.61870373 |
| PDGFB | 0.4417809 | 0.65090796 |
| PGF | 0.44114126 | 0.65090796 |
| PTX3 | 0.00048079 | 0.01995263 |
| S100A12 | 0.83079074 | 0.91703601 |
| S100A9 | 0.74576934 | 0.8518191 |
| SCG2 | 0.09675529 | 0.37643855 |
| SDC1 | 0.01066886 | 0.1562674 |
| SELE | 0.35476265 | 0.63165678 |
| SELP | 0.71623198 | 0.83776183 |
| SIRPA | 0.00533286 | 0.08852548 |
| SLAMF1 | 0.22103074 | 0.48130654 |
| SPP1 | 0.06597884 | 0.32213198 |
| TAFA5 | 0.73807269 | 0.8518191 |
| TEK | 0.66718722 | 0.8041979 |
| TGFB1 | 0.68397648 | 0.81100068 |
| TGFB3 | 0.07435962 | 0.34188154 |
| THBS2 | 0.02079153 | 0.18489615 |
| THPO | 0.00215939 | 0.04888074 |
| TIMP1 | 0.1376689 | 0.40875746 |
| TIMP2 | 0.42931178 | 0.65090796 |
| TLR3 | 0.85771407 | 0.9323993 |
| TNF | 0.01422145 | 0.16665496 |
| TNFRSF11A | 0.08941712 | 0.35773291 |
| TNFRSF11B | 0.12138282 | 0.39569001 |
| TNFRSF13B | 0.3943566 | 0.65090796 |
| TNFRSF13C | 0.49922331 | 0.68864061 |
| TNFRSF14 | 0.14186637 | 0.40875746 |
| TNFRSF17 | 0.64652502 | 0.79695411 |
| TNFRSF18 | 0.49936816 | 0.68864061 |
| TNFRSF1A | 0.01637956 | 0.16665496 |
| TNFRSF1B | 0.00046803 | 0.01995263 |
| TNFRSF21 | 0.16646155 | 0.42157218 |
| TNFRSF4 | 0.43638209 | 0.65090796 |
| TNFRSF8 | 0.60749867 | 0.77572907 |
| TNFRSF9 | 0.47634413 | 0.68560514 |
| TNFSF10 | 0.01396288 | 0.16665496 |
| TNFSF11 | 0.03400471 | 0.23642144 |
| TNFSF12 | 0.91717816 | 0.95467519 |
| TNFSF13 | 0.10982717 | 0.39177158 |
| TNFSF14 | 0.13638899 | 0.40875746 |
| TNFSF15 | 0.35036692 | 0.63165678 |
| TNFSF18 | 0.99540814 | 0.99540814 |
| TNFSF4 | 0.29443863 | 0.57277515 |
| TNFSF8 | 0.34122944 | 0.62475096 |
| TNFSF9 | 0.0008654 | 0.03078349 |
| TREM1 | 0.00264831 | 0.05495253 |
| TREM2 | 0.92400289 | 0.95467519 |
| VCAM1 | 0.14521545 | 0.40875746 |
| VEGFA | 0.08948539 | 0.35773291 |
| VEGFC | 0.17133782 | 0.42663117 |
| VEGFD | 0.43670394 | 0.65090796 |
| VSNL1 | 0.0032845 | 0.06291087 |
| VSTM1 | 0.42596032 | 0.65090796 |
| WNT16 | 0.15164513 | 0.40875746 |
| WNT7A | 0.32055783 | 0.59566344 |

**Supplemental Figure Legend**

**S1A.** Individual protein detectability percentage in all samples.

**S1B.** Frequency of protein detectability across all samples.

**S1C.** Line graph showing significant changes in expression of cytokines as in Figure 1C by patients.


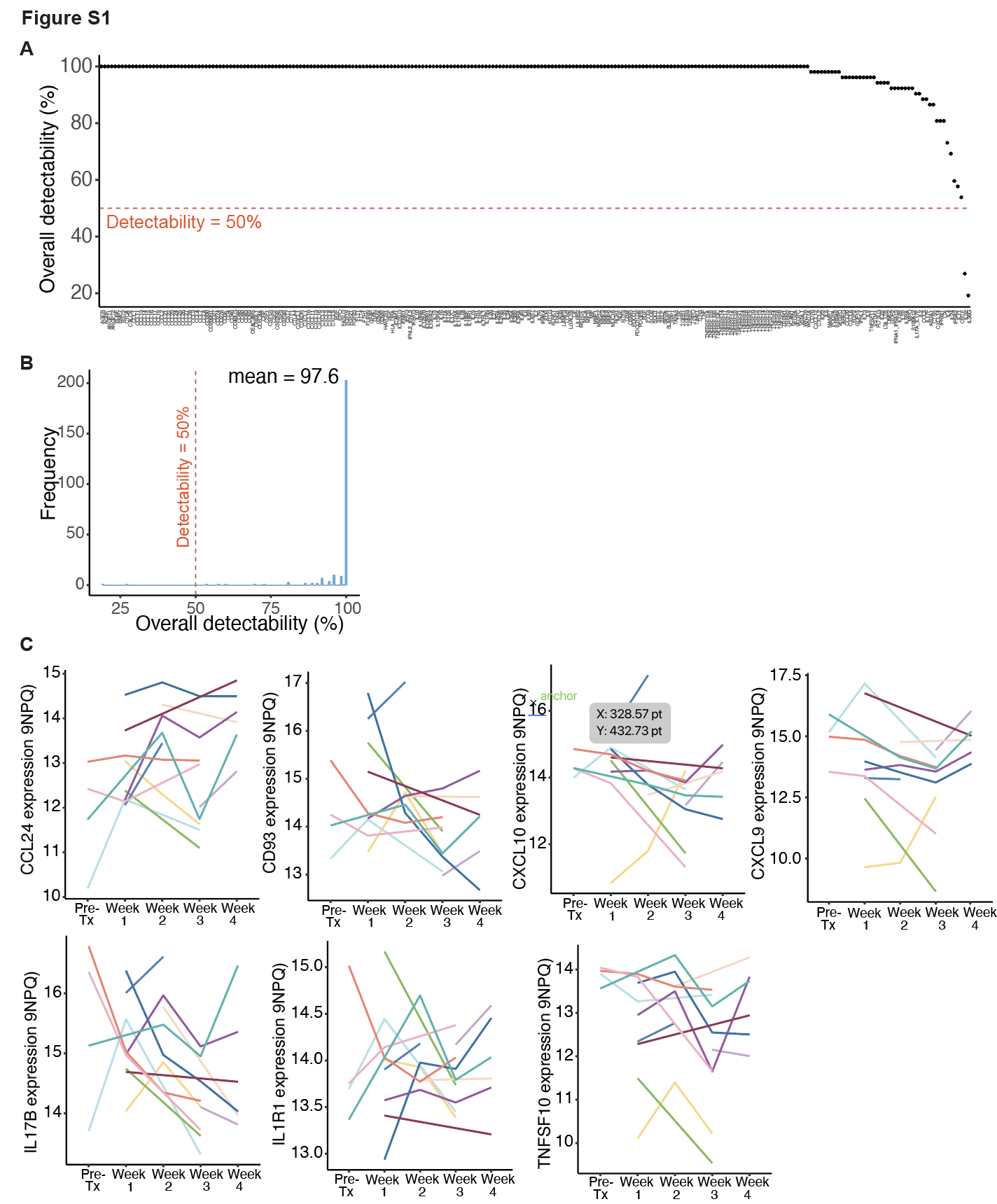

Supplement: Supplemental Tables and Figure [file mmc1.docx]
